# The Impact of BMI on Ventricular Function Recovery in Children After Pediatric Inflammatory Multisystem Syndrome (PIMS-TS)

**DOI:** 10.3390/jcm13237280

**Published:** 2024-11-29

**Authors:** Halszka Kamińska, Anna Rożnowska-Wójtowicz, Bożena Werner

**Affiliations:** 1 Department of Pediatric Cardiology and General Pediatrics, Medical University of Warsaw, 02-091 Warsaw, Poland; halszka.kaminska@wum.edu.pl; 2 Department of Pediatric Cardiology and General Pediatrics, Doctoral School, Medical University of Warsaw, 02-091 Warsaw, Poland; anna.roznowska-wojtowicz@wum.edu.pl

**Keywords:** PIMS-TS, MIS-C, BMI, echocardiography, children, recovery

## Abstract

**Objectives:** The goal of this study was to assess if body mass index (BMI) affects the pace of cardiac muscle recovery in children after Pediatric Inflammatory Multisystem Syndrome Temporally Associated with SARS-CoV-2/COVID-19 (PIMS-TS). **Methods:** A prospective single-center study enrolled consecutive children hospitalized with PIMS-TS between October 2020 and February 2022 and followed up after 6 weeks and 6 months. In all children, three-dimensional echocardiography and global longitudinal strain were used to assess ventricular function and the results were analyzed according to patients’ BMI status. **Results:** This study enrolled 170 patients aged 1–17 years, median 8.5 ± 4.43 years. Children with normal BMI (>5th and <85th percentile; *n* = 115) accounted for 67.65%, overweight and obese children (>85th percentile; *n* = 40) accounted for 23.53% and underweight children (<5th percentile; *n* = 15) accounted for 8.82% of the participants. In all patients, the means of left and right ventricular ejection fractions (LVEF and RVEF) in 3D-ECHO and average GLS were within normal limits at discharge and during follow-up. However, in children with normal weight, the function of the left ventricle improved between 6 weeks and 6 months according to both GLS and LVEF (respectively: LV GLS −20.19 ± 3.88% and −23.17 ± 2.58, *p* < 0.05; LVEF 60.68 ± 2.64% and 63.58 ± 2.49%, *p* < 0.05), while no significant improvement was observed in underweight, overweight and obese children. In patients with BMI > 85th percentile, the mean of left ventricular GLS after 6 weeks, although in the normal range, was significantly lower than in underweight children (respectively: −20.09 ± 2.5% and −23.55 ± 3.55%, *p* = 0.002), whereas left ventricle function assessed with 3D-ECHO showed no significant difference in both groups at that time (BMI > 85th percentile LVEF 61.15 ± 3.21%, BMI < 5th percentile LVEF 61.30 ± 2.71%, *p* = 0.36). During the study period, right ventricular function remained within normal limits and no significant differences according to both GLS and RVEF were reported between weight status groups. However, further significant right ventricular function improvement between 6 weeks and 6 months was observed in children with normal weight according to GLS (respectively: −22.6 ± 4.51% and −24.16 ± 2.97%, *p* = 0.02), while RVEF in 3D-ECHO remained unchanged (respectively: 64.01 ± 3.61% and 64.53 ± 3.15%, *p* = 0.63). In groups of underweight, overweight and obese children, no significant changes between 6 weeks and 6 months were observed (BMI < 5th percentile RVEF at 6 weeks 66.20 ± 2.86% and BMI < 5th percentile RVEF at 6 months 65.20 ± 2.28%, *p* = 0.58; BMI > 85th percentile RVEF at 6 weeks 63.44 ± 3.00% and BMI > 85th percentile RVEF at 6 months 64.11 ± 2.52%, *p* = 0.58). **Conclusions:** Left and right ventricular function stayed within normal limits 6 weeks after PIMS-TS regardless of patients’ BMI. Left and right ventricular function improved further between 6 weeks and 6 months after acute disease in the group of children with normal BMI. GLS is a sensitive tool for its assessment. Lower ventricular GLS in children with BMI > 85th percentile may indicate poorer left ventricular performance. Children with normal BMI may present with a more advantageous cardiac recovery pace after PIMS-TS.

## 1. Introduction

Pediatric Multisystem Inflammatory Syndrome Temporally Associated with SARS-CoV-2/COVID-19 (PIMS-TS) or Multisystem Inflammatory Syndrome in Children (MIS-C) is an aftermath of the SARS-CoV-2 pandemic [1,2,3]. The cardiovascular system is affected in the majority of patients (typically with left ventricular systolic function deterioration), although cardiac involvement is only temporary in most children.

BMI is known to affect cardiac performance, with poorer outcomes in populations of overweight and obese patients reported to date [4,5], but its impact on cardiac function recovery in patients after PIMS-TS is still unclear.

The aim of this study was to assess if and how body mass index (BMI) affects the pace of cardiac muscle recovery in children after PIMS-TS.

## 2. Materials and Methods

A prospective study was conducted in the Department of Pediatric Cardiology between October 2020 and February 2022. Consecutive patients diagnosed with PIMS-TS (according to clinical and laboratory WHO criteria) were followed up 6 weeks and 6 months after the diagnosis. In our previously published study [6], we evaluated the severity of the acute phase of the disease as a prognostic factor for cardiovascular recovery.

For the purpose of this study, patients were divided into normal weight (centile 5th–85th), overweight and obese (>85th centile) and underweight (<5th centile) groups according to the current CDC criteria.

At control points, the echocardiography was performed by two independent pediatric cardiologists, and three-dimensional echocardiography (3D-ECHO) and average global longitudinal strain (GLS) were used to assess ventricular function (Philips, Andover, MA, USA, EPIQ CVx 3D).

Left ventricular ejection fraction (LVEF) was assessed in 3D-ECHO using Philips Dynamic Heart Model (DHM), and right ventricular ejection fraction (RVEF) was assessed with the 3D Auto RV TomTec algorithm. The average longitudinal strain (GLS Avg), expressing the percentage of myocardial shortening of both ventricles, was derived from speckle tracking and post-processed using TomTec v. 2.51 software for two-dimensional apical images.

The study protocol was consistent with the standards of the Helsinki Declaration, and was approved by the University Bioethical Committee (KB/13/2021). The legal guardians and patients older than 16 years signed informed consent for participation.

Continuous data were expressed as mean and standard deviation (SD) or median and interquartile range. Normal distribution was assessed with the Shapiro–Wilk test. Differences in means between groups were assessed with the paired and unpaired *t*-test. Categorical data were expressed as percentages. Differences in proportions were compared by means of chi-square analysis (Pearson, with Yates correction when necessary). A 2-sided *p* value less than 0.05 was considered significant for all tests. Analyses were performed using the statistical package Statistica v. 13.1 software (Dell Inc., Tulsa, OK, USA).

## 3. Results

This study enrolled 170 consecutive patients aged 1–17 diagnosed with PIMS-TS and controlled in the pediatric cardiology unit. The majority of patients were male (*n* = 115) with a mean age of 8.5 ± 4.43 years. Children with a normal weight or a BMI between the 5th and 85th percentile (*n* = 115) accounted for 67.65% of the whole group, while overweight and obese children (*n* = 40) accounted for 23.53% and underweight children (*n* = 15) accounted for 8.82% of the participants.

At the time of discharge, all patients were asymptomatic with normal ventricular systolic function (LVEF and RVEF).

**Figure 1:**
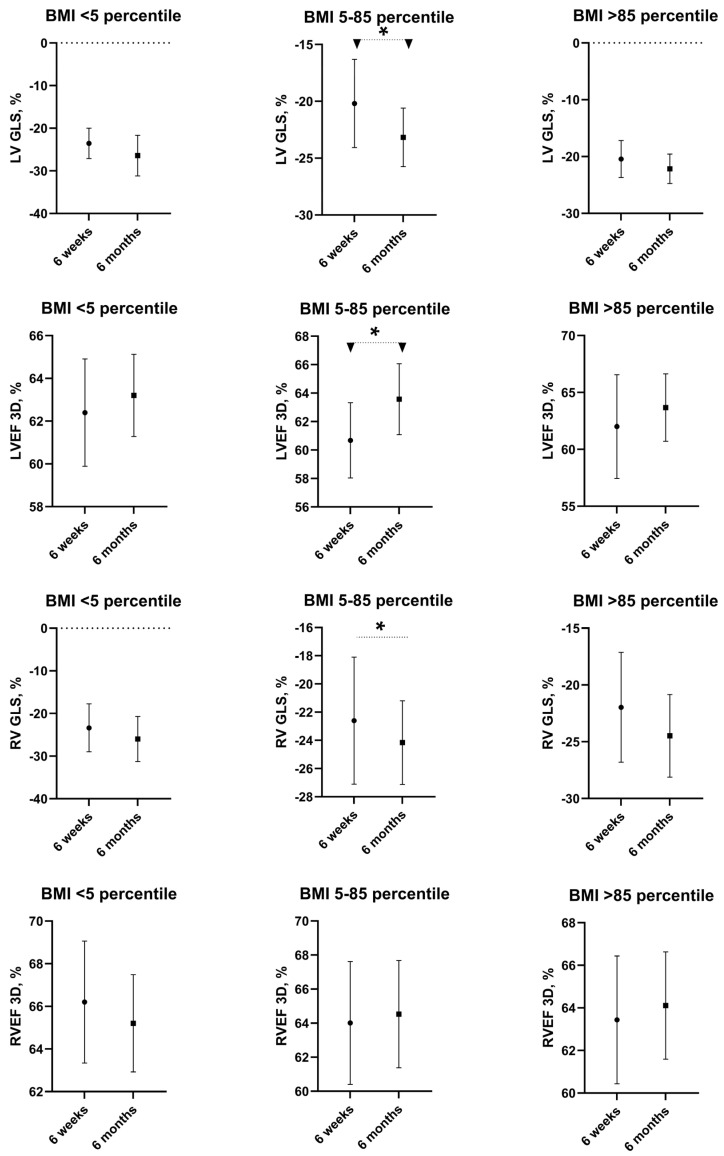
Echocardiographic parameters in underweight, normal weight, and overweight and obese children after PIMS-TS on follow-up. Values are presented as means and SD. * statistically significant difference, i.e., *p* < 0.05.

During follow-up, 130 children were evaluated after 6 weeks and 72 were evaluated again after 6 months. For all children, despite their weight status, the means of left and right ventricular ejection fraction (LVEF and RVEF) in 3D-ECHO and average GLS were reported to be within normal limits at the time of both check-up points (Table 1). However, in children with normal weight, the function of the left ventricle improved further between 6 weeks and 6 months according to both GLS and LVEF (*p* < 0.05). No significant improvement in left ventricle function was observed in underweight, overweight and obese children between 6 weeks and 6 months according to both GLS and LVEF (Table 1, Figure 1).

In patients with BMI > 85th percentile, the mean of left ventricular GLS after 6 weeks, although in the normal range, was significantly lower than in underweight children (respectively: −20.09 ± 2.5% and −23.55 ± 3.55%, *p* = 0.002), whereas left ventricle function assessed with 3D-ECHO showed no significant difference in both groups at that time (BMI > 85th percentile LVEF 61.15 ± 3.21, BMI < 5th percentile LVEF 61.30 ± 2.71, *p* = 0.36).

No significant differences between underweight, normal weight, and overweight and obese children were reported after 6 weeks and 6 months for right ventricular function according to both GLS and RVEF (Table 1). Significant right ventricular function improvement between 6 weeks and 6 months was observed in children with normal weight according to GLS (respectively: −22.6 ± 4.51% and −24.16 ± 2.97%, *p* = 0.02), but according to RVEF, right ventricular function remained unchanged (*p* = 0.63). In groups of underweight, overweight and obese children, right ventricular function, represented with both GLS and RVEF, showed no significant change after 6 weeks and 6 months (Table 1).

## 4. Discussion

In our studied group of 170 patients with PIMS-TS, a relatively small portion had abnormal BMI, with only 40 children (less than a quarter) being overweight or obese. This characteristic is in contrast to other studies involving BMI in analysis, where children with increased BMI represented significantly higher percentages of the populations. In the group of children reported by Capone et al., 45% of children were overweight or obese [7]. In smaller populations from European countries, almost half of patients had abnormally high BMI [8,9]. Our group’s nutritional status is in agreement with the characteristics of the general population of children with PIMS-TS in Poland, with a 7% obesity rate, [10,11] and is consistent with the population of Polish children in general, in which 9.1% of children have excessive BMI [12].

Interestingly, in the light of current discussion about potential pathophysiological similarities between PIMS-TS with cardiac involvement and myocarditis, the Polish population of children suffering from the latter presents with a much higher prevalence (45%) of excessive BMI [13]. The same tendency is also observed in the group of patients with COVID-19-vaccination-induced myocarditis [14].

Due to the small percentage of overweight and obese children in our studied population and the small ratio/proportion of patients with severe cardiac involvement (also typical for Polish populations; only 10 children in our group needed escalated treatment in PICU [6,10]), we were not able to establish a direct link between excessive BMI and increased severity of PIMS-TS or worse outcomes; however, this tendency was reported by Khoury et al. in an international cohort study [9].

In the studied group, underweight patients presented significantly higher means of GLS than overweight and obese patients, and therefore the results of the comparison between ventricular mechanics in children with normal and abnormal BMI were similar to those observed and reported for general populations by many authors. Kibar et al. [15] compared a group of 60 obese children aged 10–16 years old (similar to PIMS-TS patients) with healthy controls, noting significantly lower longitudinal strain values in the overweight group. Paysal et al. [5] observed that left ventricular GLS in adolescent girls was affected by BMI, with higher values reported in patients with anorexia and lower values reported in obese children (this tendency was not observed for radial and circumferential strain values).

Interestingly, our analysis demonstrated a different pace of ventricular function recovery after PIMS-TS between the children with normal and abnormal (both too low and excessive) BMI, which so far has not been noted in the literature. Even taking into account the lower numbers of underweight and overweight children in our group, they demonstrated no tendency to improve ventricular mechanics between 6 weeks and 6 months after the disease, while the means of GLS for both ventricles increased during that time in patients with normal BMI. This tendency may illustrate the higher capacity of the muscle for potential improvement in children with normal weight, although this was only visible/noticeable by GLS and not by 3D-ECHO EF. It is worth noting that the time of observed improvement overlaps with the timing of a return to “normal life” and physical activity in most children after PIMS-TS. No marked change in BMI status was apparent in our patients in contrast to the data published by Di Profio et al. [16], where the means of BMI increased during 6 months of observation after discharge.

This may reflect the fact of the positive impact of physical activity as a cornerstone of every-day life for cardiovascular outcomes, including recovery after disease impairing cardiovascular function.

In the light of these findings the restrictions of physical activity in children after PIMS-TS should be reconsidered.

As a limitation of our study, we must consider that it is a single-center study and may not accurately represent trends from the general pediatric population. The majority of studied patients had normal BMI, which makes this cohort different than those studied in many other countries. We are lacking detailed data on the diets and physical activity of underweight and overweight children during their recovery, and therefore further prospective observation is required.

## Figures and Tables

**Table 1:** Echocardiographic parameters of underweight, normal weight, and overweight and obese children after PIMS-TS on follow-up.

BMI Percentile									
	<5			5–85			>85		
	Mean	SD	*p* Value	Mean	SD	*p* Value	Mean	SD	*p* Value
LV GLS at 6 weeks, %	−23.55	3.55	0.14	−20.19	3.88	<0.0001	−20.43	3.25	0.15
LV GLS at 6 months, %	−26.42	4.76		−23.17	2.58		−22.16	2.59	
LVEF 3D at 6 weeks, %	62.40	2.51	0.53	60.68	2.64	<0.0001	62.00	4.56	0.32
LVEF 3D at 6 months, %	63.20	1.92		63.58	2.49		63.67	2.96	
RV GLS at 6 weeks, %	−23.38	5.62	0.32	−22.60	4.51	0.02	−21.97	4.84	0.26
RV GLS at 6 weeks, %	−26.00	5.30		−24.16	2.97		−24.48	3.64	
RVEF 3D at 6 weeks, %	66.20	2.86	0.58	64.01	3.61	0.63	63.44	3.00	0.58
RVEF 3D at 6 months, %	65.20	2.28		64.53	3.15		64.11	2.52	

PIMS-TS = Pediatric Multisystem Inflammatory Syndrome Temporally Associated with SARS-CoV-2/COVID-19; LVEF = left ventricular ejection fraction; LV GLS = left ventricular global longitudinal strain; RVEF = right ventricular ejection fraction; RV GLS = right ventricular global longitudinal strain.

## Data Availability

The raw data supporting the conclusions of this article will be made available by the authors on request.

## References

[1] Felsenstein S., Willis E., Lythgoe H., McCann L., Cleary A., Mahmood K., Porter D., Jones J., McDonagh J., Chieng A. (2020). Presentation, Treatment Response and Short-Term Outcomes in Paediatric Multisystem Inflammatory Syndrome Temporally Associated with SARS-CoV-2 (PIMS-TS). J. Clin. Med..

[2] Hoste L., Van Paemel R., Haerynck F. (2021). Multisystem inflammatory syndrome in children related to COVID-19: A systematic review. Eur. J. Pediatr..

[3] Santos M.O., Goncalves L.C., Silva P.A.N., Moreira A.L.E., Ito C.R.M., Peixoto F.A.O., Wastowski I.J., Carneiro L.C., Avelino M.A.G. (2022). Multisystem inflammatory syndrome (MIS-C): A systematic review and meta-analysis of clinical characteristics, treatment, and outcomes. J. Pediatr..

[4] Gherbesi E., Faggiano A., Sala C., Carugo S., Grassi G., Tadic M., Cuspidi C. (2024). Left ventricular systolic dysfunction in obesity: A meta-analysis of speckle tracking echocardiographic studies. J. Hypertens..

[5] Paysal J., Merlin E., Rochette E., Terral D., Nottin S. (2023). Impact of BMI z-score on left ventricular mechanics in adolescent girls. Front. Pediatr..

[6] Capone C.A., Subramony A., Sweberg T., Schneider J., Shah S., Rubin L., Schleien C., Northwell Health C.-R.C., Epstein S., Johnson J.C. (2020). Characteristics, Cardiac Involvement, and Outcomes of Multisystem Inflammatory Syndrome of Childhood Associated with severe acute respiratory syndrome coronavirus 2 Infection. J. Pediatr..

[7] Riphagen S., Gomez X., Gonzalez-Martinez C., Wilkinson N., Theocharis P. (2020). Hyperinflammatory shock in children during COVID-19 pandemic. Lancet.

[8] Khoury M., Harahsheh A.S., Raghuveer G., Dahdah N., Lee S., Fabi M., Selamet Tierney E.S., Portman M.A., Choueiter N.F., Elias M. (2023). Obesity and Outcomes of Kawasaki Disease and COVID-19-Related Multisystem Inflammatory Syndrome in Children. JAMA Netw. Open.

[9] Buda P., Strauss E., Januszkiewicz-Lewandowska D., Czerwinska E., Ludwikowska K., Szenborn L., Gowin E., Okarska-Napierała M., Kuchar E., Ksia̧zyk J. (2022). Clinical characteristics of children with MIS-C fulfilling classification criteria for macrophage activation syndrome. Front. Pediatr..

[10] Okarska-Napierala M., Ludwikowska K.M., Szenborn L., Dudek N., Mania A., Buda P., Ksiazyk J., Mazur-Malewska K., Figlerowicz M., Szczukocki M. (2020). Pediatric Inflammatory Multisystem Syndrome (PIMS) Did Occur in Poland during Months with Low COVID-19 Prevalence, Preliminary Results of a Nationwide Register. J. Clin. Med..

[11] Gajewska D., Harton A. (2023). Current nutritional status of the Polish population—Focus on body weight status. J. Health Inequal..

[12] Jarecka M., Kamińska H., Werner B. (2022). 10 years of myocarditis in children—A single-centre retrospective study. Pediatr. Med. Rodz..

[13] Puchalski M., Kamińska H., Bartoszek M., Brzewski M., Werner B. (2022). COVID-19-Vaccination-Induced Myocarditis in Teenagers: Case Series with Further Follow-Up. Int. J. Environ. Res. Public Health.

[14] Kaminska H., Roznowska-Wojtowicz A., Cacko A., Okarska-Napierala M., Kuchar E., Werner B. (2023). Three-Dimensional Echocardiography and Global Longitudinal Strain in Follow-Up After Multisystem Inflammatory Syndrome in Children: Six-Month, Single-Center, Prospective Study. J. Pediatr..

[15] Kibar A.E., Pac F.A., Ece I., Oflaz M.B., Balli S., Bas V.N., Aycan Z. (2015). Effect of obesity on left ventricular longitudinal myocardial strain by speckle tracking echocardiography in children and adolescents. Balk. Med. J..

[16] Di Profio E., Leone A., Vizzuso S., Fiore G., Pascuzzi M.C., Agostinelli M., Dilillo D., Mannarino S., Fiori L., D’Auria E. (2023). Longitudinal Anthropometry and Body Composition in Children With SARS-CoV-2-Associated Multisystem Inflammatory Syndrome. J. Pediatr. Gastroenterol. Nutr..

